# Incidence and risk factors of deep venous thrombosis following arthroscopic posterior cruciate ligament reconstruction

**DOI:** 10.1097/MD.0000000000007074

**Published:** 2017-06-02

**Authors:** Dongyang Chen, Qiangqiang Li, Zhen Rong, Yao Yao, Zhihong Xu, Dongquan Shi, Qing Jiang

**Affiliations:** a Department of Sports Medicine and Adult Reconstructive Surgery, Nanjing Drum Tower Hospital Affiliated with the Medical School of Nanjing University, Nanjing Jiangsu; b Department of Sports Medicine and Adult Reconstructive Surgery, Nanjing Drum Tower Hospital, Clinical College of Nanjing Medical University, Nanjing; c Laboratory for Bone and Joint Diseases, Model Animal Research Center, Nanjing University, Nanjing, Jiangsu; d Department of Orthopedics, The Third Affiliated Hospital of Soochow University, Suzhou, China.

**Keywords:** arthroscopy, deep venous thrombosis, posterior cruciate ligament, thromboprophylaxis, venography

## Abstract

The objective of this study was to identify the incidence and associated risk factors for deep venous thrombosis (DVT) after arthroscopic posterior cruciate ligament (PCL) reconstruction.

This study included 128 patients who underwent arthroscopic PCL reconstruction. Venography was performed on the operated leg 3 days postoperatively. The patients were divided into 2 groups based on whether they had DVT. A correlation analysis was performed to determine the factors associated with DVT.

Of all the 128 patients, 28 patients (21.9%) developed DVT, with 4 (3.1%) in a proximal location. Significant differences were found in the mean age, time of application of tourniquet, mean VAS scores, mean d-dimer level, mean cholesterol level, and various surgical procedures in patients with DVT compared with those without DVT. DVT is difficult to diagnose solely based on clinical symptoms.

The incidence of DVT was 21.9% in patients who underwent arthroscopic PCL reconstruction. The rate of asymptomatic clots in the calf region was rather high after PCL reconstruction, and the rate of proximal clots was 4%. Older age, longer durations of tourniquet application, higher VAS scores and D-dimer levels, and complex surgical procedures were all substantial risk factors for DVT after PCL reconstruction. The treatment of DVT with batroxobin and anticoagulants was effective and safe.

## 1 Introduction

Venous thromboembolism (VTE), which includes deep venous thrombosis (DVT) and pulmonary embolism (PE), is a common complication in orthopedics. Nevertheless, DVT is regarded as a relatively rare complication in knee arthroscopy. The incidence of DVT after knee arthroscopy has been reported to range from 1.5% to 17.9%,^[1–6]^ and the necessity of using anticoagulant remains controversial. The American College of Chest Physicians (ACCP) does not recommend the use of anticoagulants after arthroscopy in patients without any risk factor for VTE.^[7]^ However, a strong indicator of the need for routine thromboprophylaxis includes findings of high incidences of DVT, which has been reported in 8% to 41.2% of patients after arthroscopically assisted anterior cruciate ligament (ACL) reconstruction.^[3,8,9]^ Moreover, several cases of fatalities from PE after ACL reconstruction have been reported.^[10,11]^ Posterior cruciate ligament (PCL) reconstruction operations carry a greater risk of developing DVT, as it is a more invasive operation than ACL reconstruction. The total complication rate was the highest for PCL reconstruction (20.1%), followed by ACL reconstruction (9.0%), meniscectomy (2.8%), meniscal repair (7.6%), and chondroplasty (3.6%).^[12]^ Dong et al^[13]^ also found a 20.5% incidence of DVT among PCL reconstruction patients who did not receive anticoagulants.

However, there has been no study addressing DVT in PCL reconstruction patients with routine venography, which is the criterion standard for DVT diagnosis. The purpose of this study was to identify the incidence of and associated risk factors for DVT after arthroscopic PLC reconstruction with routine venography and to provide a clinical reference. We hypothesized that the incidence of DVT after PCL reconstruction, detected with venography, would be relatively high without anticoagulant drugs.

## 2 Materials and methods

We conducted a retrospective analysis of 181 patients who had undergone reconstruction of PCL, PCL combined with ACL, and/or medial and/or lateral collateral ligament (ACL/MCL/ LCL) between December 2007 and February 2015. All the patients experienced poor function of the knee joint. Patients were excluded if they had a history of VTE, had previously undergone surgery for popliteal vein injuries or anticoagulation or with fracture in the lower extremity, or if there was any contraindication to contrast venography.

All patients received general anesthesia. A thigh tourniquet inflated to 270 mmHg was applied to the operated leg. It was deflated if the procedure lasted >90 minutes and was reinflated 10 minutes later if necessary. The single-bundle technique combined with autohamstring tendon reconstruction was performed for PCL reconstruction by the same surgeon. Anticoagulant drugs were not used in any patient postoperatively. Pneumatic compression was applied for 30 minutes twice per day in all patients after surgery. Progressive exercises for quadriceps began 24 hours after the operation. Patients were mobilized until discharge if they were not diagnosed with DVT.

The rising temperature and redness of the skin around the knee, calf tenderness (Neuhof test), and pain in the calf on dorsiflexion (Homan test) were examined and recorded. A unilateral venography^[14]^ was performed on the operated leg 3 days postoperatively in all patients. All patients received a venography examination the day before surgery to exclude preoperative DVT. DVT was diagnosed if a constant intraluminal filling defect was seen on 2 sequential films. All venograms were performed by 1 technician and reviewed by 2 experts blinded to the clinical status of the patients. Any thrombus involving the popliteal, femoral, or iliac veins was defined as a proximal DVT. All other thrombi were classified as distal DVTs. Patients diagnosed with DVT received thrombolytic therapy by using batroxobin together with low-molecular-weight heparin (LMWH). Only those with proximal DVT were restricted to the bed. The dose of batroxobin was 5 Brabender Units 2 to 3 times for distal DVT and 3 to 5 times for proximal DVT every other day. LMWH was injected subcutaneously at a dose of 30 mg once daily. Color Doppler ultrasound was performed every 3 days for patients who had proximal DVT to observe the thrombolysis outcome. The patients were discharged from the hospital when the thrombosis disappeared.

All participants provided written informed consent to the experimental procedures, which were approved by the medical ethics committee of the Nanjing University and in accordance with the Declaration of Helsinki.

The proportions of patients with DVT and proximal DVT were calculated. The mean or frequencies of the factors such as age, sex, body mass index (BMI), type of surgery (PCL reconstruction alone or combined with ACL/MCL/LCL), smoking history, serum markers including prothrombin time (PT), activated partial thromboplastin time (APTT), thrombin time (TT), international normalized ratio (INR), fibrinogen (FIB), d-dimer level, platelet count (PLT), cholesterol level and triglyceride (TG) level, visual analogue scale (VAS) scores, and durations of both operations and applications of the tourniquets, and comorbidities including hypertension, diabetes, cardiovascular diseases, history of venous thromboembolism, and history of malignancy were compared between patients with DVT and without DVT to determine the associated high-risk factors of DVT. Data were compared between the 2 groups using Student *t* tests and *χ*^2^ tests. We conducted a multivariable logistic regression analysis to further analyze the risk factors for DVT following arthroscopic PCL reconstruction. All statistical analyses were performed using SPSS Statistics software, version 19.0 (IBM, Armonk, NY).

## 3 Results

A total of 181 patients underwent PCL reconstruction during the 7-year study period. Fifty-three patients were excluded for the following reasons: preoperative DVT (25 patients), concurrent fracture in the lower extremity (20 patients), and an age younger than 18 years (8 patients). Thus, 128 patients were finally enrolled in this study, comprising 92 males and 36 females, with a mean age of 38.4 ± 12.8 years and a mean BMI of 24.0 ± 3.7 kg/m^2^.

Of all the 128 patients, 28 patients (21.9%) developed DVT (Table 1). Proximal DVT was detected in 4 patients (3.1%). Among the distal cases, 9 involved ≥2 veins and 7 were close to the popliteal vein. Among the proximal cases, 2 were in the femoral vein, and the remaining 2 were in the popliteal vein. Thirteen of the 28 patients were asymptomatic. None of the patients presented with symptoms of PE, and none of them died. After thrombolytic therapy with batroxobin and LMWH, none of the 28 patients showed signs of PE, and all 4 proximal DVTs had disappeared. Additionally, there was no fatal bleeding or bleeding in a critical organ in the patients under thrombolytic therapy. One patient developed minor wound bleeding, indicated by wound ecchymosis.

**Table 1:**
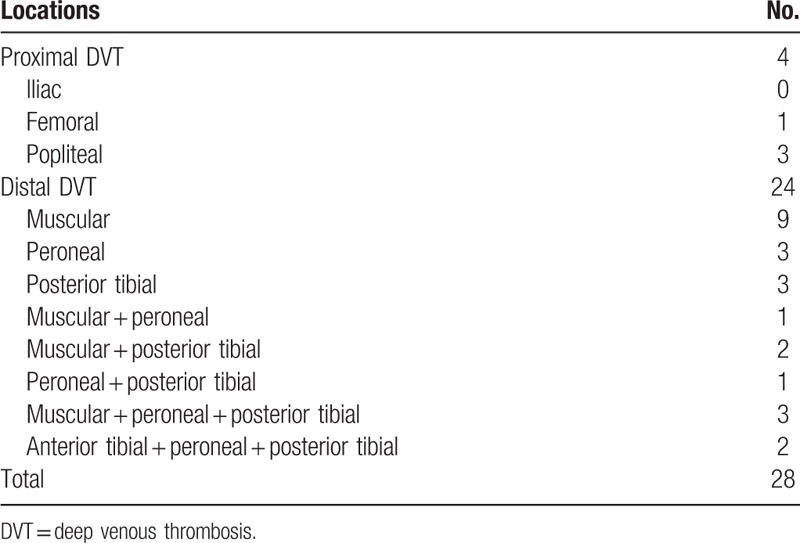
The locations of DVT.

The mean durations of surgery and tourniquet application were 115.0 ± 31.3 minutes and 75.1 ± 15.7 minutes, respectively. Several potential risk factors for DVT were compared and analyzed (Table 2). BMI, sex, operation time, smoking history, PT, APTT, TT, INR, FIB, TG, and PLT were not significant risk factors for DVT. However, the mean age, duration of tourniquet application, mean VAS score, mean d-dimer level, and mean cholesterol level were significantly higher in those with DVT than those without DVT. Additionally, the incidence of DVT after PCL reconstruction combined with ACL/MCL/LCL reconstruction was significantly higher than the incidence of DVT after PCL reconstruction alone. Moreover, after adjusting for age, duration of tourniquet, VAS scores, d-dimer level, and cholesterol level (Table 3), type of surgery (odds ratio = 2.25, *P* = .012) was found to be related to postoperative DVT.

**Table 2:**
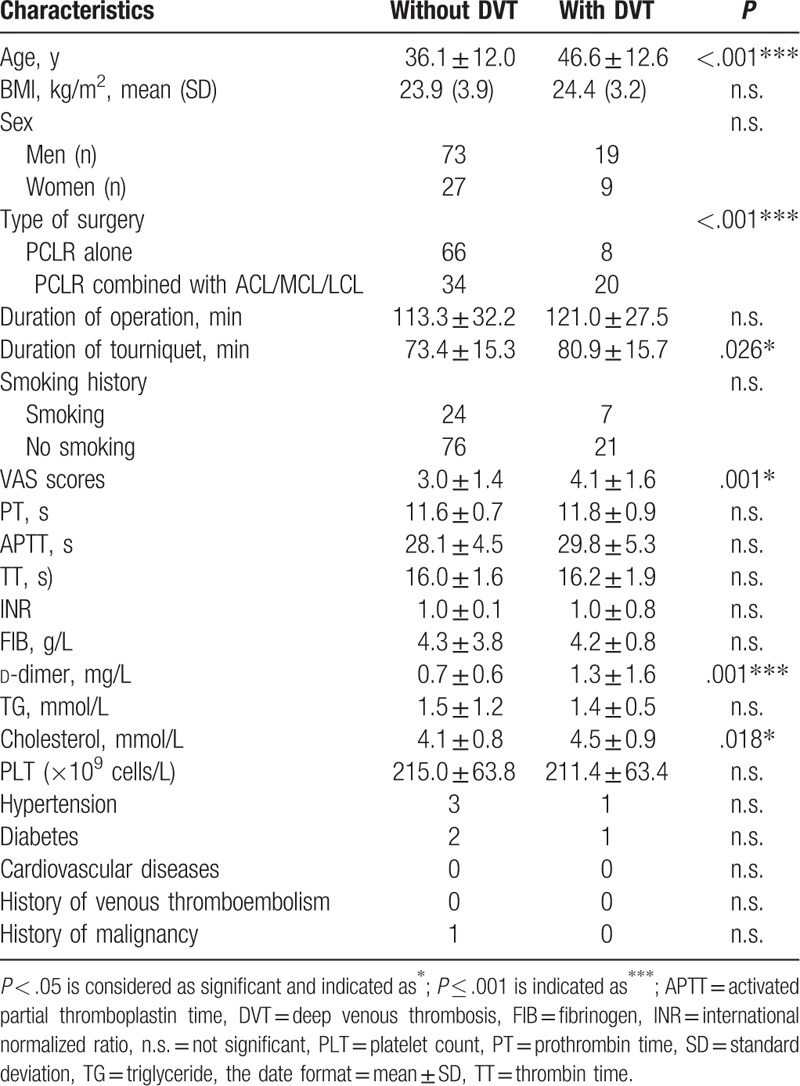
Clinical characteristics of the patients with or without DVT.

**Table 3:**
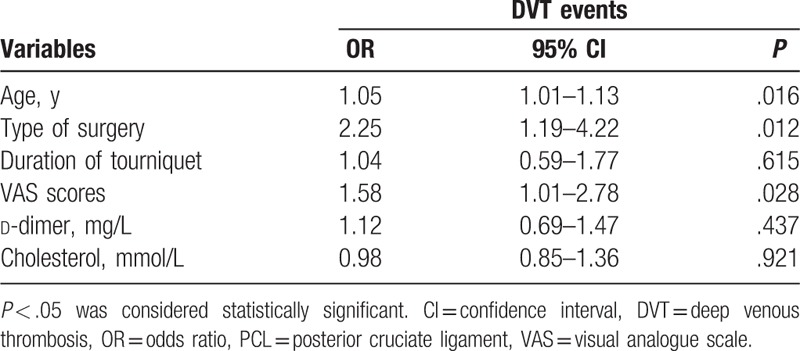
Multivariable analysis of the risk factors for DVT following arthroscopic PCL reconstruction.

## 4 Discussion

A 21.9% incidence of DVT and a 3.1% incidence of proximal DVT were observed on the third day after arthroscopic PCL reconstruction in the absence of an anticoagulant drug. DVT is a common complication after major orthopedic surgery. However, little literature is available regarding the incidence of DVT after arthroscopic surgery. Some authors have recommended against using anticoagulant drugs in patients with no risk factors for DVT after arthroscopic surgery based on the reported low incidences of DVT of 1.7% to 5.7%.^[1,5,15,16]^ However, some reported incidences have been higher, at 7.2% to 41.2%, after arthroscopic ACL reconstruction, which is strongly suggestive of a need for routine thromboprophylaxis.^[3,8,9,13,17]^

Of the 128 patients, 28 patients (21.9%) developed DVT. Proximal DVT was detected in 4 patients (3.1%). Among the distal DVT cases, 9 involved ≥2 veins, and 7 were close to the popliteal vein. According to the 9th Guideline of ACCP, DVTs involving multiveins in the calf veins are at risk for extending to the popliteal vein.^[7]^ Although there is a lower risk of embolism compared with proximal DVT, in some studies, calf DVT is associated with a high rate of fatal emboli.^[18–20]^ Approximately 25% of thrombi were reported to propagate to the tibial and popliteal veins without treatment.^[20]^

Arthroscopic cruciate ligament reconstructions, especially PCL reconstruction, are more invasive and require longer operations and tourniquet applications compared with routine arthroscopic operations.^[21]^ It is likely that having undergone a complex surgical procedure is a strong risk factor for DVT. The need for anticoagulants to prevent DVT after arthroscopic ACL reconstruction remains controversial. Collision et al^[1]^ reported a very low incidence of DVT after ACL reconstruction of approximately 1.5%, which is similar to the incidence of 1.78% reported by Adala et al.^[16]^ However, both of these studies used ultrasonography to diagnose DVT, which is not sensitive enough to diagnose asymptomatic DVT. It has been reported that approximately 40% to 50% of DVT cases are asymptomatic.^[22]^ The incidence of our study was more reliable because of the use of venography, which had a high sensitivity and specificity for the diagnosis of both symptomatic and asymptomatic DVT, even for small thromboses in calf veins.^[19]^ Some authors have reported that LMWH is an appropriate anticoagulant for decreasing the DVT rate after arthroscopic surgery.^[4,6,14]^ PCL reconstruction is more invasive than ACL reconstruction. The knee must be repeatedly flexed and extended to allow the tendon to pass through the tunnel, which can cause extensive tissue damage around the knee and increase the opportunity for the formation of DVT. Dong et al^[13]^ reported a 17.4% incidence of DVT after PCL reconstruction alone and a 7% higher incidence for those PCL reconstruction combined with other ligament reconstruction, which was confirmed in our study. The high incidence can be attributed to the need for a longer duration of tourniquet use (*P* = .026) and extra-articular incisions, which can cause severe pain (*P* = .001) and reduce activity, both of which are risk factors for DVT following multiligament reconstruction (*P* < .001). Moreover, PCL reconstruction remained the risk factor for DVT following multivariable analysis. For this reason, we suggest routine anticoagulant use for preventing DVT after arthroscopic PCL reconstruction, especially in multiligament reconstruction.

Metabolic syndrome (MS), with a worldwide reported prevalence ranging from 7% to 56%,^[23]^ has been reported to be a significant risk factor for DVT.^[24,25]^ However, in our study, MS components, including abdominal obesity, hypertension, and hyperglycemia, did not show significant differences between patients with DVT and those without DVT. This finding can be partially ascribed to the fact that patients in this study had a young mean age (38.4 ± 12.8 years).

The tourniquet is indispensable for achieving a good surgical field. However, tourniquets can impede blood reflux and cause tissue hypoxia in the lower extremities, resulting in accumulation of acidic metabolites, which can stimulate venous tunica intima and induce a state of hypercoagulation. It has been reported that patients who had a tourniquet placed during total knee arthroplasty were more likely to develop DVT than those who did not.^[26]^

PCL fractures were often caused by high-energy trauma, which could also cause extensive tissue damage around the knee, including the venous tunica intima. Therefore, some of the patients develop arthralgia and arthrocele preoperatively. Operations are a major cause of pain and swelling. The use of a tourniquet and extra-articular incisions could lead to these results.^[12]^ Additionally, prolonged use of physiological saline can lead to an engorged knee, following the compression of the veins around the knee, resulting in blood flowing more slowly. It is clear that 15 of the 28 patients with DVT presented with these 2 symptoms, but there were more patients who presented with these symptoms in those without DVT. DVT is difficult to diagnose based on clinical symptoms, and an auxiliary image examination should be performed to aid in its diagnosis. Venography, as the standard recommended screening method for DVT, demonstrated an ability to yield accurate and reliable results.

Aging is regarded as a weak risk factor for DVT.^[27]^ Sun et al^[17]^ reported that patients older than 35 years were at a high risk for experiencing DVT, which could be because of increased immobility and decreased exercise following ACL reconstruction.

Higher cholesterol levels had not been addressed with regard to DVT after arthroscopic surgery. In our study, the mean cholesterol level in the group of patients with DVT was significantly higher than the mean level in those without DVT. It is likely that a higher cholesterol level could increase blood viscosity and decrease blood flow, leading to a high risk of DVT.

Batroxobin has been confirmed as a safe thrombolytic for the treatment of acute DVT after arthroplasty and arthroscopy.^[17,28]^ In our study, none of the patients developed symptomatic PE during thrombolysis, and batroxobin's effect on thrombolysis was significant. Furthermore, no vena cava filter was applied during treatment. Although there were a few cases of DVT, the achievement concerning PE was great. Regarding the small sample size, the efficacy of thromboprophylaxis on fresh clots in the below knee region still remains uncertain.

A high VAS score indicates a high risk of DVT. In this study, the mean VAS score in patients with DVT was significantly higher than the mean level in those without DVT. This result is possibly because of the inactive rehabilitation exercise and reduced range of motion and frequencies of ankle pumping caused by the severe postoperative pain.

d-dimer is a marker of endogenous fibrinolysis, which is widely used in screens for DVT.^[29]^ However, the plasma d-dimer assay is a sensitive but less specific marker of DVT^[29,30]^ because many conditions such as infection, inflammation, trauma, hemorrhage, and renal insufficiency can cause a high value of d-dimer.^[31–33]^ In our study, a higher value of d-dimer was detected in subjects with DVT compared to those without DVT.

There were some limitations in our study. The sample size was relatively small, and the clinical importance of our results would be stronger with a larger sample size. Our result is likely lower than the true overall incidence because we may have missed contralateral thrombosis with unilateral venography. AD-dimeritionally, all the examinations were performed on the patients who remained in the hospital, so we did not know whether someone developed DVT after hospitalization. Furthermore, we did not perform follow-up of the distal DVTs owing to the invasiveness and high cost of venography.

## 5 Conclusions

The incidence of DVT was 21.9% among patients who underwent arthroscopic PCL reconstruction. The rate of asymptomatic clots in the calf region is rather high after PCL reconstruction, and the rates of proximal clots or symptomatic thromboembolic events are limited. Older age, longer tourniquet application times, higher VAS scores and d-dimer levels, and more complex surgical procedures were all significant risk factors for DVT after PCL reconstruction. Thus, thromboprophylaxis is advocated in these patients.
